# Bioactive Natural Compounds for Therapeutic and Nutraceutical Applications in Neurodegeneration

**DOI:** 10.3390/nu14112216

**Published:** 2022-05-26

**Authors:** Maria Antonietta Panaro, Chiara Porro

**Affiliations:** 1 Department of Biosciences, Biotechnologies and Biopharmaceutics, University of Bari, 70125 Bari, Italy; 2 Department of Clinical and Experimental Medicine, University of Foggia, 71121 Foggia, Italy; chiara.porro@unifg.it

Figure 1 summarizes the neuroprotective effects played by bioactive compounds examined in this Special Issue. New perspectives and insights into the therapeutic and nutraceutical applications of the bioactive natural compounds finalized the prevention and treatment of neurodegenerative diseases. The inflammation of the Central Nervous system (CNS), known as neuroinflammation, is an important feature in the pathogenesis and progression of neurodegenerative diseases.

Neuroinflammation is characterized by microglia activation with the consequent production of high quantity of the pro-inflammatory cytokines and mediators leading to increasing neuronal cell death. Over the past 20 years, there are more and more studies confirming the protective and preventive action of some foods, defined as nutraceuticals, on the state of health and wellbeing in general. Lately, much research has focused on the role of food components that are active in the prevention of neurodegenerative diseases, which are now constantly increasing.

In this editorial for MDPI Nutrients Special Issue (SI) “Bioactive Natural Compounds for Therapeutic and Nutraceutical Applications in Neurodegeneration”, we report the recent advances concerning the mechanisms of action of nutritional compounds in neurodegenerative and neurological conditions.

Currently, preventive, or therapeutic treatments for combating neurodegenerative diseases are limited. In addition, the improvement in life expectancy determines an increase in the incidence of neurodegenerative diseases, including Alzheimer’s and Parkinson’s disease: Aging is in fact the most important risk factor for these incurable diseases [1,2]. Decreased neuroinflammation can be obtained by using natural compounds, including flavonoids known to modulate inflammatory responses [3,4,5,6].

Lee et al., reported the effects of Erinacine A-enriched *Hericium erinaceus* mycelia (EAHEM) in protecting against neurological disorders, reducing oxidative stress in the brain, and further preventing chronic inflammation, thereby decreasing amyloid aggregation and improving learning and memory. In fact, it seems that feeding EAHEM to mice can drastically reduce the expression of iNOS in the brain, which implies that its protective effect is determined through a direct reduction in oxidative stress and inflammation [7].

It was found following the acute phase of ischemia and in the period following it with neurodegenerative characteristics and mechanisms such as those observed in Alzheimer’s disease (AD). In this regard, myricetin, a flavonoid that was first identified in Myricaceae plants about 2 centuries ago, could be a promising complementary agent in the future against the development of post-ischemic brain neurodegeneration due to its pleiotropic properties, including anti-amyloid, anti-phosphorylation of tau protein, and anti-inflammatory, antioxidant, and autophagous properties, as well as increasing acetylcholine and, in addition, its ability to effectively cross BB [8].

The pyramidal neurons’ death in the hippocampus with progressive brain atrophy are typical changes for post-ischemic brain neurodegeneration and for AD. In fact, research indicates that, after ischemia, the brain may develop the typical neurodegeneration observed in AD, as post-ischemic brain damage causes selective neuronal death in the hippocampus typical of AD with progressive brain atrophy. Neuroinflammatory changes play a key role in the progression of post-ischemic brain neurodegeneration [9,10]. A direct consequence of ischemic damage is, both in animal models and in humans, the accumulation of amyloid in the form of amyloid plaques and neurofibrillary tangles. In recent years, curcumin has received a lot of attention as a possible candidate for the prevention and therapy of neurodegeneration due to its pharmacological effects, being a molecule with multiple targets and that is of versatile use and with a reduced risk of resistance to therapy, carrying out an anti-inflammatory, antioxidant, antiamyloid, and anti-dementia action [11,12]. Low bioavailability is one of the best-known limiting factors for the use of curcumin in the clinical field; therefore, future research must focus on defining the therapeutic potential of curcumin in the treatment of neurodegenerative diseases [13].

Among the flavonoids, quercetin has also been described for its multiple pharmacological applications, including anti-inflammatory, antitumor, anti-apoptotic activity, and for its neuroprotective abilities by acting on the functional responses of microglial cells with different mechanisms, preserving BBB’s integrity. The ability of quercetin to influence microRNA expression represents an interesting aspect in the regulation of inflammation and immune responses. This flavonoid regulates the extent of miRNAs involved in the advancement of AD and normalizes gene and miRNA expression in the hippocampus of diabetic rats in an impaired learning pattern. The bioavailability of quercetin can be increased by different drug delivery systems: for example, by encapsulating it in natural exosomes and making it more efficient as a potential therapeutic tool for AD therapy [14].

It is known that neurotoxic amyloid β plays a fundamental role in AD pathogenesis than BACE1, an enzyme involved in the production and amyloid deposition, which represents a target for AD prevention. In this regard, sulforaphane (isothiocyanate-4- (methylsulfinyl) -butane), a sulfur-rich compound found in cruciferous vegetables, including broccoli and cabbage, is a well-known chemoprotective compound, which has also been shown to have a variety of biological activities including antioxidant, antidiabetic, anti-inflammatory, and neuroprotective effects. Youn et al. (2021) in an analysis based on kinetics and computational studies reported that sulforaphane showed six-times stronger selective inhibitory activity against BACE1 than other known compounds, such as resveratrol and quercetin, making sulforane a promising novel candidate with potent and selective inhibitory properties of BACE1, which play an important role in the prevention of AD [15].

Lactoferrin (LF) is an 80 kDa iron-binding multifunctional glycoprotein belonging to the transferrin family contained in various cells, including neurons and microglia. In addition to acting as a vehicle, LF itself possesses protective effects during neuropathological conditions, such as degenerative disorders. In AD, LF expression increases significantly within cortical neurons and is present in amyloid deposits. LF is increased in surviving dopaminergic neurons within the substantia nigra pars compacta (SNpc) in Parkinson’s disease (PD) patients and PD mouse models [16]. While on the one hand, the accumulation of LF in the brains of patients with PD and AD may be the consequence of a dysregulation of iron homeostasis; on the other hand, it could be hypothesized that the LF in the brain can counteract neurodegenerative processes, thus playing a protective role towards neurons [16].

In the study reported by Ryskalin et al., LF significantly attenuates the amount of cell loss and mitochondrial alterations produced by methamphetamine (METH). Furthermore, LF can attenuate the dissipation of autophagy-related proteins, which is massively induced by METH [17]. However, the specificity of LF as a neuroprotector refers to the specific cellular phenotype. The induction of autophagy is the key to targeting non-METH toxicity, which is not observed in non-DA cell lines. In fact, in this study, LF Reduces METH-Induced Toxicity in PC12 Cells, whereas METH toxicity is not detected in U87MG cells, in which cell differentiation rather than neuroprotection is observed. This phenotype-dependent variability confirms that LF has antiproliferative and experimental and differentiating properties by promoting molecular pathways in ubiquitous biochemical processes that acquire different relevances depending on the specific cell type (i.e., neuroprotective or cytostatic effects) [17].

Many studies have proposed that a reduction in the function of mitochondria is one of the causes in the development of PD. PD disease leads to the loss of dopaminergic neurons of the substance nigra, and this loss appears to be due to mitochondrial damage. Currently, treatment or treatment to combat PD is limited. Recently, it has recently been demonstrated by Jhuo et al. (2020), in an MPP+-Induced SH-SY5Y cell model of PD, that teaghrelin alleviates mitochondrial dysfunction and apoptosis. Teaghrelin is a compound originating from Chin-Shin Oolong tea, exhibiting ghrelin agonist activity. The ghrelin axis and ghrelin receptor are involved in the preservation of dopaminergic neurons with potential implications in PD treatment [18].

Since teaghrelin can activate the AMPK/SIRT1/PGC-1α and ERK1/2 pathways to antagonize MPP+-induced cell death, this result suggests that teaghrelin may be a suitable candidate for the therapeutic treatment of PD [18].

Amyotrophic lateral sclerosis (ALS) is an incurable chronic progressive neurodegenerative disease with progressive muscular paralysis reflecting the degeneration of motor neurones in the primary motor cortex, corticospinal tracts, brainstem, and the spinal cord. The review of Polina S. Goncharova (2021) after having analysed 39 studies, including seven meta-analyses, reports that the inclusion of vitamins and a ketogenic diet is the most significant protector of ALS development, reducing the process of motor neuron degeneration and slowing down the rate of disease progression [19]. However, there are several limitations in this thematic research. The intake of different nutrients is likely to have a variable effect on the progression of ALS; moreover, men and women respond differently to nutrients in ALS. Thus, further studies are needed to study the gender effects of nutrients in monotherapies and polytherapies, the role of the microbiota in nutrient synthesis, and the role of nutrigenetics in nutrient absorption, transport, accumulation, metabolism, and excretion.

In the review research study, Popescu et al. (2021) investigated the relationship between Vitamin K2 (VK2) and the following aspects in AD: Aβ neurotoxicity, neuroinflammation, mitochondrial dysfunction, cognition, cardiovascular health, dysbiosis, and AD comorbidities. Overall, these studies tend to suggest a pivotal role for VK2 in the prevention and treatment of AD. A surprising aspect is that despite the presence of this research highlighting a clear link between VK2 and AD, there are still no human clinical studies available after investigating this relationship [20].

Kraft et al., in their paper analysed the effects of treatment with trehalose, a disaccharide found in bacteria, yeasts, insects, fungi, and plants but not in mammals in a mouse model of neuropathic pain in terms of nociception, motor functions, physiology, spatial and social behaviour, activity, learning, and memory [21]. Prolonged administration to mice reduced nociceptive hypersensitivity after sciatic nerve injury but adversely affected overall activity and learning performance, suggesting a negative impact of trehalose on alertness or alertness [21]. In conclusion, although dietary supplements can reduce chronic pain, they may interfere with alertness.

Spirulina is a filamentous, spiral-shaped blue algae, belonging, more precisely, to the Cyanobacteria class (cyanobacteria), and it contains a series of components, i.e., large quantities of proteins; vitamins A and E of group B; and mineral salts such as iron, magnesium, calcium, and phosphorus that make it an ideal food. Thanks to these characteristics, it can protect against free radicals and the damage they cause to the body (premature aging and neurodegenerative diseases). Trotta et al., in a systematic review summarized the latest findings on the neuroprotective role of Spirulina, its positive effects on the activation of glial cells and on the treatment of neurodegenerative diseases, particularly PD, AD, and Multiple Sclerosis. Several lines of evidence testify for peculiar neuroprotection mechanisms, including antioxidant and anti-inflammatory activities in the brain parenchyma, which make Spirulina a potential pharmacological agent in the prevention and treatment of neurodegenerative diseases [22]. However, despite the numerous and encouraging scientific evidence both in in vitro and in vivo models, further studies are needed to better clarify the mechanisms of action of Spirulina.

## Figures and Tables

**Figure 1:**
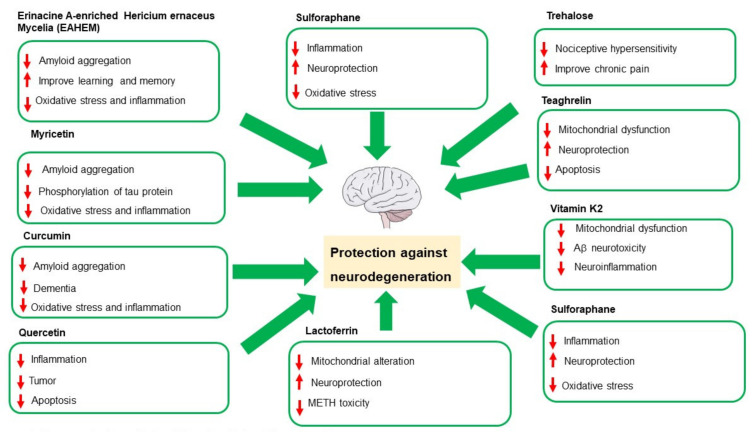
Neuroprotective effects of Bioactive Natural Compounds.
